# Buffalo Medical and Surgical Journal

**Published:** 1871-07

**Authors:** 


### Editorial.

### Buffalo Medical and Surgical Journal.

We announce the completion of our tenth volume with some feelings of personal pride as well as pleasure. Those of our readers who commenced with us, and understand the circumstances of our beginning, will fully appreciate the triumph of completing ten years of editorial labor without having had to "change base" or even "halt." It will be remembered by them, how our *Journal* was born during the darkest period of our late war, though perhaps they may not all know that it took several years to breath into it the breath of independent life, so that it could really be said to be a "living soul." It was received kindly by the editorial brotherhood with hearty good wishes, always attended by the sad prediction that at such a time of financial and political distress, such enterprise could not prove successful; their own journals were about to be suspended, and the idea of a new one being offered to the medical public appeared to them inconceivable. It was so well supported (by the proprietor) that it did not suffer in the least by the general distress, and was for a long time, the only medical journal in the State, except the reprints. At length it became able to sustain independent life, and has gradually grown in strength and influence until it has completed its tenth year, passing that uncertain and perilous age during which so many of its associates fall by the way. It owes its life, "under a favoring providence," to the general and hearty support of the profession, and while it has received much from contributors and subscribers, we believe it has rendered ample *quid pro quo.*

It has been conducted upon the most liberal principles of exact justice to all; its pages open for the expression of intelligent, honest conviction, however much it might differ from the views of the editor, or of the profession in general. It has been the policy to allow the fullest expression of personal opinion, knowing that the attentive readers of the journal are able to gather for themselves the wheat, and leave the chaff, (if there is any,) "to be burned in unquenchable fire." It cannot be supposed that everything we publish meets our approval, and no one will hold the editor responsible for the opinions of others.

Our readers will be more anxious to learn the prospects of the future, than to read the history of the past. They cannot but have observed how distinguished members of the profession in distant places are favoring us with their contributions, and thus greatly adding to the value of our journal. It cannot have escaped careful observers that every year has increased the number and merit of our original articles, and that the pages of the journal contain in original and republished papers, a complete compend of the progress of practical medicine and surgery: such facts speak more favorably of our future, than anything we can promise.

Again, our list of subscribers is a source of pride which cannot be overlooked. Within the legitimate circle of the journal, the members of the profession favor it with their names, and the exceptions to this are only sufficient to prove the general rule, these few would not increase, very much, our satisfaction, if added. The journal has, a few times in ten years, been tardy a day or two in its appearance. This was not owing to any delay in the payment of the dues of subscribers, but to unavoidable accidents in the office of publication.

We had proposed, at this period, should we ever reach it, to rest from our editorial labors, and let our works follow us, but we have become so joined to our idols that as the period arrives, we cannot let them go. We should be lost to life, if, after the dnties of the day, we *must* not spend the evening and often the early morning hours in professional converse with our old friends, who have so faithfully and so generously sustained us. It is a matter of *pride* and of *pleasure* that makes us promise our readers ten years more of medical journal. Alas! the uncertain future. We will offer the profession an impartial medium of communication while we may, and when we cannot longer bear its labors, hope to transmit it to abler, but never to more earnest hands.

